# Assessment of Interprofessional Competencies in Primary Health Care: A Scoping Review

**DOI:** 10.1111/tct.70491

**Published:** 2026-08-13

**Authors:** Ana Caroline Machado, Alessandra da Silva Kulkamp, Ana Paula Rocha, Ana Paula Veber, Fernanda Manzini, Isabel Cardoso de Carvalho, Mareni Rocha Farias, Marina Raijche Mattozo Rover, Marselle Nobre de Carvalho, Silvana Nair Leite

**Affiliations:** ^1^ Postgraduate Program in Pharmaceutical Services and Policies Federal University of Santa Catarina Florianópolis Santa Catarina Brazil; ^2^ Department of Public Health State University of Londrina Londrina Paraná Brazil

**Keywords:** interprofessional collaborative practice, interprofessional competencies, interprofessional education, primary health care, self‐assessment

## Abstract

**Background:**

Interprofessional education (IPE) and interprofessional collaborative practice (IPCP) are essential strategies for developing the necessary interprofessional competencies (IPC) for collaborative practice. While self‐assessment tools measuring IPC are widely used in educational settings, their application in primary health care (PHC) is limited. This study aimed to identify and analyse the available tools for assessing IPC in PHC, highlighting their characteristics and applicability.

**Methods:**

A scoping review was conducted by searching eight databases. Data extracted included publication details and descriptions of assessment tools.

**Results:**

The search identified 981 records, of which 21 studies describing 15 different assessment tools were included. Most studies were published between 2016 and 2024, conducted primarily in the United States, and involved professionals from Nursing, Medicine and Pharmacy. The tools were mainly based on the Interprofessional Education Collaborative (IPEC) Core Competencies and the Canadian Interprofessional Health Collaborative frameworks, underscoring their established role as key references for IPC. Common assessed domains and competencies included roles and responsibilities, teamwork and collaboration and interprofessional communication. Additionally, some tools incorporated competencies related to community centred care, collaborative leadership and public health, indicating their potential relevance to PHC.

**Conclusion:**

This review provides a comprehensive overview of the IPC assessment tools used in primary health settings, offering insights into the diversity of evaluation methods. Faculty, health professionals and researchers should identify the most appropriate tool according to the context in which it will be used, ensuring its applicability and the generation of results that will contribute to the enhancement of IPE and IPCP.

## 1 Background

Primary health care (PHC)‐based health systems are grounded in equity, social justice and the right to health [1]. The essential principles for maintaining such a system include the ability to respond equitably and efficiently to the health needs of citizens, the capacity to monitor progress for continuous improvement and the implementation of intersectoral interventions. The benefits of health systems supported by high‐quality PHC are internationally recognised, yet persistent health inequalities remain a critical challenge for policymakers [2].

Interprofessional collaborative practice (IPCP) has been proposed as a strategy to address the challenges by integrating the competences and complementary skills of health and social professionals to improve care and optimise resources [3, 4, 5, 6, 7]. Studies have shown positive effects of teamwork in healthcare settings, including improved continuity and coordination of care and beneficial changes in patient outcomes. In contrast, the lack of collaboration among healthcare professionals can lead to misdiagnosis, fragmented care and higher mortality rates [8, 9].

Recognising these benefits, health educators are increasingly encouraged to integrate interprofessional education (IPE) into practice‐based settings. The growing implementation of interprofessional programmes in PHC has shown positive results for both patients and students [10]. In this context, interprofessional competencies (IPC) aim to guide the training and continuous development of healthcare professionals to strengthen IPE and IPCP, ultimately supporting health system goals and improving health outcomes [4]. IPC are described as the complex integration of knowledge, skills, attitudes, values and judgement that enable healthcare professionals to apply these components in collaborative situations, aiming to improve health outcomes equitably [11, 12].

Translating these competencies into practice involves their effective implementation alongside organisational and situational factors. As IPC evolves, assessment techniques must be either capable of capturing this dynamism or undergo constant revision. Ideally, evaluation should be conducted through direct observation of performance in interprofessional teams, allowing for an assessment of real‐world performance in complex interactions with colleagues and patients [13].

A wide range of methods and tools are used to assess IPE students and evaluate programmes, combining qualitative and quantitative approaches [14]. Students should be able to provide various forms of evidence demonstrating their IPC, whether for educational requirements, residency programmes or employment. However, systematically observing IPC in authentic settings remains a challenge [15, 16, 17, 18, 19].

Recent literature suggests that while IPC assessment tools are widely used in diverse educational and health settings, their applicability in PHC contexts remains largely unexplored [20, 21]. Generic IPC tools designed for hospitals or specialised clinical settings often fail to capture the specific contextual and behavioural aspects of PHC, the first point of contact between health systems, communities and social determinants of health [22]. This highlights the need to establish specific standards for training and evaluation within PHC [23]. This scoping review aimed to address the need by identifying the tools available for assessing IPC in healthcare students and professionals within the PHC setting and describing their characteristics, including the evaluation methods and theoretical foundations.

*Generic IPC tools designed for hospitals or specialised clinical settings often fail to capture the specific contextual and behavioural aspects of PHC, the first point of contact between health systems, communities and social determinants of health*.

## 2 Methods

### 2.1 Study Design

This scoping review followed the Preferred Reporting Items for Systematic reviews and Meta‐Analyses extension for Scoping Reviews guidelines (PRISMA‐ScR) and the Joanna Briggs Institute (JBI) Manual for Evidence Synthesis [24, 25, 26]. The protocol was registered within the Open Science Framework (https://osf.io/3gph5/).

### 2.2 Eligibility Criteria

Inclusion and exclusion criteria were established using the population, concept and context (PCC) method to identify studies that employed tools to assess interprofessional or collaborative competencies (Concept) in health students and professionals (Population), within the setting of PHC (Context) [25].

Articles utilising quantitative, qualitative and mixed methodologies were considered eligible for inclusion in the analysis. Studies including undergraduate and graduate students, as well as healthcare professionals, were included. Different assessment strategies were also considered, such as questionnaires, scales, instruments, tools, rubrics, checklists and surveys, provided they assessed IPC.

Reviews, books, grey literature and nonempirical studies (e.g., editorial comments and opinions) were not considered eligible for inclusion.

### 2.3 Information Sources

The search strategy was conducted by searching eight databases: PubMed via Medical Literature Online (MEDLINE), Scopus, Web of Science, Scientific Electronic Library Online (SciELO), Literature of Latin America and the Caribbean (LILACS), Cumulative Index to Nursing and Allied Health Literature (CINAHL), Excerpta Medica Database (Embase) and Google Scholar. Additionally, further information about the tools was obtained from other data sources, such as manuals, websites and references cited in the selected articles.

### 2.4 Search Strategy

The search strategies were developed using the library services of the Universidade Federal de Santa Catarina (see Supporting Information Appendix A) based on Health Sciences Descriptors (DeCS), Medical Subject Headings (MeSH) and keywords frequently used in studies on the same topic, identified through the authors' preliminary reading (Table 1). No limits for period or language were applied.

**TABLE 1:** Keywords.

Themes	Keywords
Theme 1	‘core competenc*’ OR ‘interprofessional competenc*’ OR ‘collaborative competenc*’ OR ‘competências interprofissionais’ OR ‘competências colaborativas’ OR ‘habilidades interprofesional*’ OR ‘habilidades colaborativas’
Theme 2	assessment* OR evaluation OR measurement OR tool OR instrument OR scales OR scaling OR test* OR rate OR ratings OR survey* OR questionnaire* OR Instrumentos OR instrumento OR ferramenta OR escalas OR teste OR testes OR cuestionarios OR questionários OR avaliação OR evaluación
Theme 3	‘Primary Health Care’ OR ‘Primary Healthcare’ OR ‘Primary Care’ OR ‘Atenção Básica à Saúde’ OR ‘Atención Primaria de Salud’

### 2.5 Selection Process

An initial search was conducted in the Medical Literature Analysis and Retrieval System Online (MEDLINE), the International Prospective Register of Systematic Reviews (PROSPERO) and the Cochrane Database of Systematic Reviews, and the Joanna Briggs Institute (JBI) Evidence Synthesis, and no review with the same purpose was identified.

After removing duplicates using Mendeley Desktop 1.19.8, two researchers independently screened the studies using Rayyan, following the inclusion and exclusion criteria [27]. Any disagreements were discussed until consensus was reached. The reference lists of the studies analysed and previous similar reviews were examined to identify eligible studies not found in the search.

Various PHC settings were considered, including simulation centres. Studies were excluded if they did not assess competencies, if they assessed competencies but did not use an assessment tool, if the experience was not interprofessional (involving at least two different healthcare professions) or if the experiences did not occur in PHC settings or simulations of PHC. However, while the experience had to be interprofessional, the assessment study could be conducted with only one profession.

### 2.6 Data Charting Process

Data extraction was carried out independently by two researchers using a table in Excel. Whenever necessary, the extraction tables were reviewed and updated. Disagreements were resolved by consensus within the research group.

### 2.7 Data Items

The following data were extracted: characteristics of studies (author, year, country, journal, purpose, setting, participants and the assessment tool) in Table 2 and characteristics of the tools (title, validation status, description and number of items) in Table 3. The assessed domains and competencies were primarily based on the lead authors' descriptions, which generally aligned with specific competency frameworks. Given the variability in nomenclature, terminological standardisation was established for this review. Due to the unavailability of the CanMEDS 2009 framework, a deductive categorisation of the Health Professional Collaborative Competency Perception Scale (HPCCPS) items was conducted. Since this mapping relied on the original authors' descriptions, it is potentially subject to interpretative or labelling bias.

**TABLE 2:** Characteristics of the sources of evidence.

Authors, year, country, journal	Purpose	Setting	Participants	Methodology type	Assessment tool
Sevin et al. (2016); United States; American Journal of Pharmaceutical Education	Investigate the effect of an interprofessional service‐learning course on health professions students' self‐assessment of Interprofessional Education Collaborative (IPEC) competencies	Interprofessional student‐run free clinic	Nursing, social work and pharmacy students	Quantitative nonrandomised	Interprofessional Education Collaborative (IPEC) Competency Self‐Assessment Survey
Widyandana (2018); Indonesia; The Indonesian Journal of Medical Education	Evaluate students interprofessional attitudes during the implementation of IPE in a community‐based program	Community and Family Health Care (CFHC) program	Medical, nursing and nutrition students	Quantitative descriptive	Interprofessional Attitude Scale (IPAS)
Hartnett et al. (2019); United States; Journal of Dental Education	Evaluate the effectiveness of an interprofessional paediatric dental health clinical placement in developing interprofessional competencies	Paediatric dental and/or paediatric primary care setting	Medical, nursing and dental students	Quantitative nonrandomised	Interprofessional Collaborative Competency Attainment Survey (ICCAS)
Randita et al. (2019); Indonesia; Journal of Multidisciplinary Healthcare	Investigate the effect of a community‐based interprofessional learning on the development of collaborative competencies	Community health centres	Medical and midwifery students	Quantitative nonrandomised	Indonesian version of the Interprofessional Collaborator Assessment Rubric (ICAR) instrument
Tonarelli et al. (2020); Italy; Acta Biomed for Health Professions	Validate the Italian version of the Chiba Interprofessional Competency Scale (CICS29)	Various settings, including primary healthcare	Nursing, medicine, physical therapy, rehabilitation, psychiatry, radiology, psychology, social work, clinical analysis and education professionals	Quantitative descriptive	Italian version of the Chiba Interprofessional Competency Scale (CICS29)
Gilliam et al. (2020); United States; Currents in Pharmacy Teaching and Learning	Evaluate the design of an interprofessional introductory pharmacy practice experience (IP‐IPPE)	Federally qualified health centre (FQHC) serving underserved population	Pharmacy students	Mixed methods	Interprofessional Collaborative Competencies Attainment Survey (ICCAS)
Johansson et al. (2020); United States; Journal of Medical Education and Curricular Development	Evaluate an interprofessional rural rotation in the development of interprofessional and public health competencies	Local health department and primary care setting	Dental, imaging science, public health, medical, nursing, pharmacy and PhD students	Quantitative descriptive	Untitled questionnaire
Soemantri et al. (2020); Indonesia; Journal of Interprofessional Care	Validate an Indonesian version of the Chiba Interprofessional Competency Scale (CICS29) and evaluate the students' interprofessional competencies after completing an interprofessional education course	Primary healthcare centres in the local community	Medical, dental, public health, nursing and pharmacy students	Quantitative descriptive	Indonesian version of the Chiba Interprofessional Competency Scale (CICS29)
Chen et al. (2021); United States; Journal of Interprofessional Education & Practice	Describe and evaluate a longitudinal and interprofessional curriculum for undergraduate and graduate students at a geriatric medical centre	Geriatrics primary care	Geriatric, integrated geriatrics and palliative care, pharmacy, social work and law students	Mixed methods	Interprofessional Collaborative Competency Attainment Survey (ICCAS)
Diniz et al., (2021); Brazil; Revista Ciência Plural	Analyse the collaboration performed by professionals from the family health strategy and the expanded family health and primary care centre, as well as describe the work process developed and identify the collaborative competencies in the development of this practice	Primary Healthcare Units	Nursing, nutrition and physical therapy professionals	Qualitative	Untitled questionnaire
Gruss and Hasnain (2021); United States; Journal of Interprofessional Education & Practice	Describe the structure, process and results of the evaluation of the first year of implementation of a community‐based course	Community‐based care setting	Medical, nursing, pharmacy, applied health sciences, public health and social work students	Mixed methods	IPECC‐SET 38 tool
Timm and Schnepper (2021); United States; Journal of Interprofessional Care	Evaluate a new clinical education model, focused on interprofes‐ sional practice and service to the community, in effectively provide clinical training for students	Community‐based healthcare model for underserved populations	Nursing, social work, physical education and educational counselling students and faculty	Mixed methods	Interprofessional Education Collaborative (IPEC) Competency Self‐Assessment Survey
Ernawati et al. (2022); Indonesia; Journal of Interprofessional Education & Practice	Evaluate the validity and reliability of the Indonesian Collaboration Competence Questionnaire (ICCQ) and the Intercultural Sensitivity Scale translated (ISS)	Primary health care centres and hospitals	Physicians, nurses, dentists, midwives and pharmacists/pharmacy technicians professionals	Quantitative descriptive	Indonesian Collaboration Competence Questionnaire (ICCQ)
Lunde et al. (2022); Norway; Advances in Simulation	Explore healthcare students' experiences with common and subacute patient scenarios in primary care and their role in developing interprofessional collaborative competence	Simulated interprofessional primary care scenarios	Medical, advanced geriatric nursing master's and bachelor's nursing students	Mixed methods	Norwegian version of the Interprofessional collaborative competency attainment survey (ICCAS)
Miselis et al. (2022); United States; Journal of Interprofessional Care	Report the impact of interprofessional education in longitudinal primary care practices on students' interprofessional knowledge, attitudes, skills and behaviours	Family medicine clinics	Nutrition, social work, medical and physician assistant students	Mixed methods	Interprofessional Collaborative Competency Attainment Survey (ICCAS) e Review of Interprofessional Competencies (RIPC)
Goto and Haruta (2023); Japan; Journal of General and Family Medicine	Clarify differences in self‐assessment of interprofessional competency in Japan based on profession and facility type	Various settings in primary healthcare	Healthcare providers from medicine, public health nursing, nursing, pharmacy, rehabilitation therapy, care management, social work, psychiatric social work and care work	Quantitative descriptive	Japanese Self‐assessment Scale of Interprofessional Competency (JASSIC)
Haruta and Goto (2023); Japan; Journal of Interprofessional Care	Explore factors associated with interprofessional competencies among healthcare professionals in Japan	Various settings in primary healthcare	Healthcare providers from medicine, public health nursing, nursing, pharmacy, rehabilitation therapy, care management, social work, psychiatric social work and care work	Quantitative descriptive	Japanese Self‐assessment Scale of Interprofessional Competency (JASSIC)
Waltz et al. (2023); United States; Journal of Interprofessional Education & Practice	Describe and evaluate a primary care simulation to improve interprofessional competencies	Telehealth primary care simulation	Athletic training, nursing, optometry, osteopathic medicine, pharmacy and physical therapy students	Quantitative descriptive	Interprofessional Collaborative Competency Attainment Survey‐Revised (ICCAS‐R)
Zeien et al. (2023); United States; Journal of Interprofessional Care	Evaluate the impact of interprofessional relationships among volunteers in the street medicine program through their perception of competency development and interprofessional behaviours	Street medicine program	Medicine, nursing, occupational therapy, physical therapy, public health, social work and undergraduate prehealth volunteers	Quantitative nonrandomised	Interprofessional Collaborative Competencies Attainment Survey (ICCAS)
Haruta and Goto (2024); Japan; BMJ Open	Explore factors associated with healthcare professionals' subjective perceptions of complex issues in primary care settings in Japan	Various settings in primary healthcare	Healthcare providers from medicine, public health nursing, nursing, pharmacy, rehabilitation therapy, care management, social work, psychiatric social work and care work	Quantitative descriptive	Japanese Self‐assessment Scale of Interprofessional Competency (JASSIC)
Peranson et al. (2024); Canada; Journal of Chiropractic Education	Describe the results of evaluating a module on interdisciplinary management of low back pain and highlight the factors that contributed to its successful implementation	Academic family health team in an inner‐city primary care teaching site	Medicine, chiropractic, physiotherapy, pharmacy, nursing, nurse practitioner, occupational therapy, physiotherapy assistants and occupational therapist assistants students	Quantitative nonrandomised	Health Professional Collaborative Competency Perception Scale (HPCCPS)

**TABLE 3:** Characteristics of the tools.

Assessment tool	Validation status	Description	Number of items
Interprofessional Collaborative Competency Attainment Survey (ICCAS)	Content validation, construct validity through exploratory factor analysis and internal consistency	Self‐assessment survey on changes in competencies related to interprofessional collaboration in students and professionals before and after IPE training interventions. It uses a 7‐point Likert scale (*strongly disagree* to *strongly agree*) and N/A option.	20
Norwegian version of the Interprofessional collaborative competency attainment survey (ICCAS)	Cross‐cultural adaptation, internal structure and reliability	Norwegian version of the Interprofessional collaborative competency attainment survey (ICCAS). Self‐assessment survey on changes in competencies related to interprofessional collaboration in students and professionals before and after IPE training interventions. It uses a 5‐point Likert scale (*strongly disagree* to *strongly agree*) and N/A option.	20
Interprofessional Collaborative Competency Attainment Survey‐Revised (ICCAS‐R)	Internal consistency validation and paired *t*‐tests	Revised version of the Interprofessional collaborative competency attainment survey (ICCAS). Self‐assessment survey on changes in competencies related to interprofessional collaboration in students and professionals before and after IPE training interventions. It uses a 7‐point Likert scale (*strongly disagree* to *strongly agree*) and N/A option.	20
Japanese Self‐assessment Scale of Interprofessional Competency (JASSIC)	Validation process including expert discussion, cognitive debriefing, statistical analysis of internal consistency and concurrent/convergent validity	Self‐assessment Scale for individual professionals to assess interprofessional competencies, particularly in the primary care setting. A 6‐factor structure comprising 18 items, with three items per factor. Each item is rated on a 5‐point Likert scale (*not applicable* to *highly applicable*), with a total score range of 18 to 90. Higher scores indicate a more positive self‐assessment of interprofessional competency.	18
Interprofessional Education Collaborative (IPEC) Competency Self‐Assessment Survey	Content validation, construct validity via exploratory and confirmatory factor analyses and internal consistency	Self‐assessment survey to evaluate individual student competencies related to collaborative practice in a healthcare degree program. Each item is rated on a 5‐point Likert scale (*strongly disagree* to *strongly agree*).	42
Health Professional Collaborative Competency Perception Scale (HPCCPS)	Face and content validity, temporal stability and responsiveness	Self‐assessment scale, premodule and postmodule, to measure changes in self‐perceived confidence to collaborate in practice. It is an 8‐item scale with an additional construct (9th item) on the postsession application that asks about overall global change. Responses are rated on a 5‐point Likert scale (0–10 rating; *not at all very confident* to *very confident*).	9
Indonesian Collaboration Competence Questionnaire (ICCQ)	Content validity (expert review), pilot test, construct validity via Confirmatory Factor Analysis (CFA) and internal consistency reliability	Self‐assessment questionnaire measuring collaborative competencies in Indonesian healthcare professionals. With three factors of collaborative competencies across 28 statements. Responses are rated on a 5‐point Likert scale (*strongly disagree* to *strongly agree*). The maximum possible score is 140 and the level of collaborative competency is determined by the mean score. Respondents scoring above the mean are classified as having high competence.	28
Italian version of the Chiba Interprofessional Competency Scale (CICS29)	Cross‐cultural adaptation, factorial validity, convergent validity and internal consistency	Italian version of the Chiba Interprofessional Competency Scale (CICS29). A self‐assessment scale measuring collaborative awareness and practical interprofessional collaboration skills. Rated on a 5‐point Likert scale (*disagree* to *agree*), it is suitable for longitudinal studies, including preintervention and postintervention comparisons or assessments among individuals with similar backgrounds	29
Questionnaire by Johansson et al. (2020)	Lacks formal psychometric validation	Self‐assessment pre–post‐test to evaluate changes in students' knowledge of interprofessional team and public health competencies. Students rate their knowledge in each domain using a 4‐point Likert scale (*not at all knowledgeable* to *very knowledgeable*).	15
Questionnaire by Diniz et al., 2021	Lacks formal psychometric validation	Self‐assessment questionnaire about collaborative competencies, consisting of 6 domains, each with three statements, for a total of 18 statements. It uses a 5‐point Likert scale (*strongly agree* to *strongly disagree*). Each domain is scored between a minimum of 3 points and a maximum of 15 points. The presence of each domain is classified as follows: present, 12 to 15 points; latent, 8 to 11 points; absent, 3 to 7 points	18
Review of Interprofessional Competencies (RIPC)	Lacks complete psychometric validation, but preliminary assessment demonstrated internal consistency reliability	Self‐assessment survey designed to measure proficiency in IPEC subcompetencies in trainees. Trainees rate their proficiency on a 5‐point Likert scale (*novice* to *expert*).	30
Indonesian version of the Interprofessional Collaborator Assessment Rubric (ICAR) instrument	Translation and back‐translation, inter‐rater reliability and internal consistency reliability	Indonesian version of the Interprofessional Collaborator Assessment Rubric (ICAR), a competency‐based assessment rubric designed for health and social students and providers. It can be used by faculty by observation and by students for self‐assessment. The rubric includes a 4‐point scale with behavioural descriptors (minimal to mastery), a ‘Not Observable’ option and a comment field for concrete behavioural examples.	29
Indonesian version of the Chiba Interprofessional Competency Scale (CICS29)	Cross‐translation and back‐translation, construct validity via Confirmatory Factor Analysis (CFA) and internal consistency	Indonesian version of the Chiba Interprofessional Competency Scale (CICS29). A self‐assessment scale measuring collaborative awareness and practical interprofessional collaboration skills. Rated on a 5‐point Likert scale (*disagree* to *agree*), it is suitable for longitudinal studies, including preintervention and postintervention comparisons or assessments among individuals with similar backgrounds	29
Interprofessional Attitude Scale (IPAS)	Content assessment for coverage and clarity, construct validity via exploratory and confirmatory factor analyses and internal consistency	Self‐assessment scale designed to assess students' perceptions of interprofessional competencies. It includes five subscales and uses a 5‐point Likert (*strongly disagree* to *strongly agree*).	27
IPECC‐SET 38 tool	Content validation based on IPEC core competencies, construct validity and reliability	Self‐efficacy tool for competence in interprofessional collaborative practice. The competencies are divided into four sections, each competency is rated on a 100 point scale (no confidence at all to totally confident).	38

### 2.8 Synthesis Methods

**FIGURE 1:**
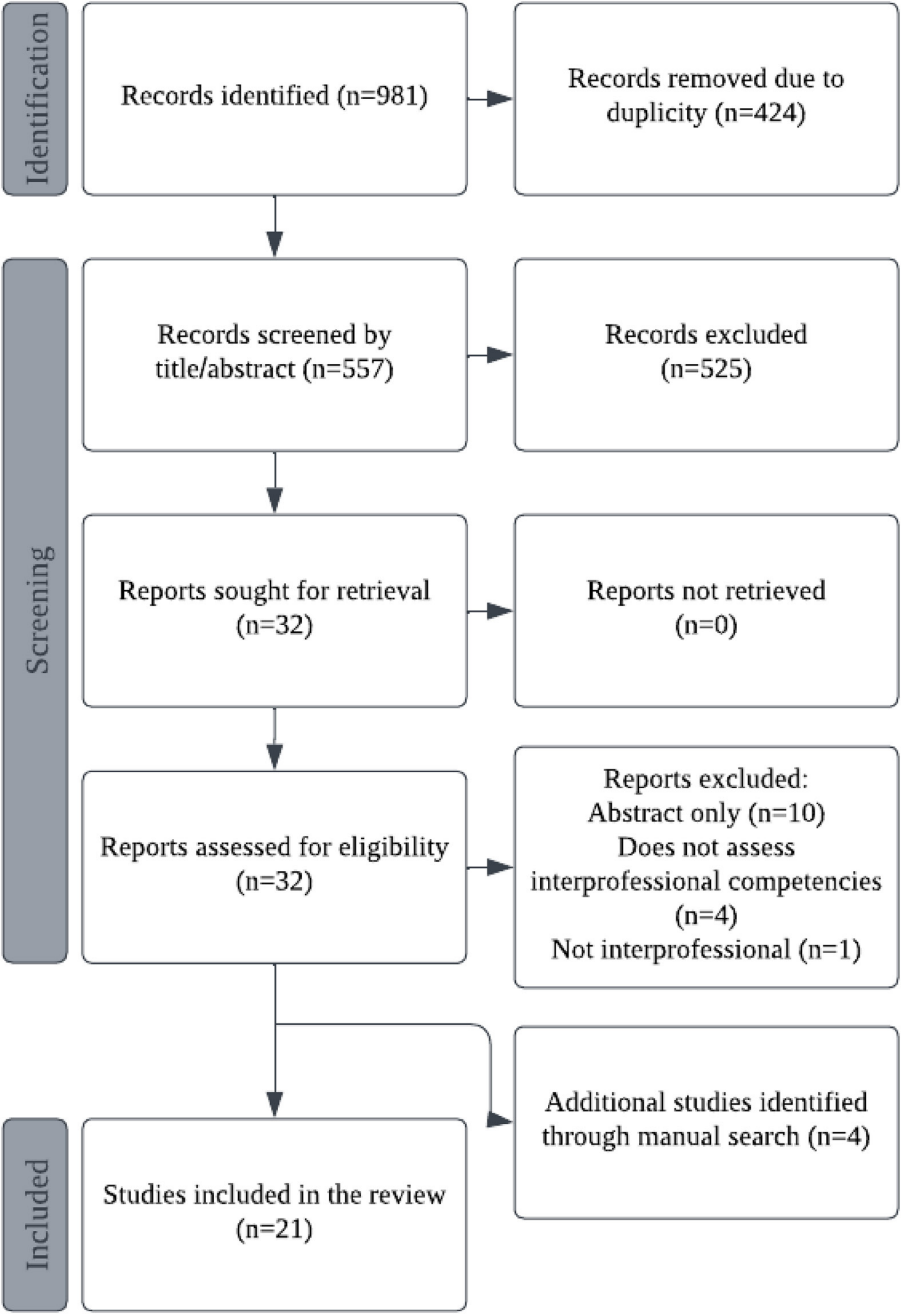
PRISMA‐ScR flowchart.

The selection process is presented using the PRISMA‐ScR flowchart (Figure 1). Data extraction results from the studies, and tools are presented through quantitative and qualitative synthesis, using tables and highlighting key themes such as the evaluation methods, the theoretical foundations and the domains and competencies assessed.

### 2.9 Quality Appraisal

We evaluated the quality of the included articles using the Mixed Method Appraisal Tool (MMAT), version 2018. Although formal quality assessment is not mandatory for scoping reviews, the MMAT was employed as a descriptive tool to enhance the interpretability of our findings and provide transparency regarding the evidence base [28, 29]. The appraisal was performed independently by two researchers, with discrepancies resolved by consensus. In accordance with MMAT guidelines, each study was assessed against five criteria specific to its research design. Each criterion was rated as ‘Yes’ if met, ‘No’ if not met or ‘Cannot tell’ if the study lacked sufficient information for a clear judgement. As the MMAT discourages the calculation of a single overall quality score, we have presented the detailed results of the appraisal for each criterion in Table 4.

**TABLE 4:** Quality appraisal.

Quantitative nonrandomised studies	Are the participants representative of the target population?	Are measurements appropriate regarding both the outcome and intervention (or exposure)?	Are there complete outcome data?	Are the confounders accounted for in the design and analysis?	During the study period, is the intervention administered (or exposure occurred) as intended?
Sevin et al. (2016)	CT	Y	Y	N	Y
Hartnett et al. (2019)	Y	Y	Y	N	Y
Randita et al. (2019)	CT	Y	Y	N	Y
Zeien et al. (2023)	CT	Y	CT	N	Y
Peranson et al. (2024)	N	Y	N	N	Y
Quantitative descriptive studies	Is the sampling strategy relevant to address the research question?	Is the sample representative of the target population?	Are the measurements appropriate?	Is the risk of nonresponse bias low?	Is the statistical analysis appropriate to answer the research question?
Widyandana (2018)	Y	CT	Y	CT	Y
Tonarelli et al. (2020)	Y	CT	Y	CT	Y
Johansson et al. (2020)	CT	CT	N	Y	Y
Soemantri et al. (2020)	Y	Y	Y	Y	Y
Ernawati et al. (2022)	Y	CT	Y	CT	Y
Goto and Haruta (2023)	Y	N	Y	CT	Y
Haruta and Goto (2023)	Y	N	Y	CT	Y
Waltz et al. (2023)	Y	Y	Y	Y	Y
Haruta and Goto (2024)	Y	N	Y	CT	Y
Mixed‐methods studies	Is there an adequate rationale for using a mixed‐methods design to address the research question?	Are the different components of the study effectively integrated to answer the research question?	Are the outputs of the integration of qualitative and quantitative components adequately interpreted?	Are divergences and inconsistencies between quantitative and qualitative results adequately addressed?	Do the different components of the study adhere to the quality criteria of each tradition of the methods involved?
Gilliam et al. (2020)	Y	Y	Y	CT	Y
Chen et al. (2021)	Y	Y	Y	Y	Y
Gruss and Hasnain (2021)	Y	Y	Y	CT	N
Timm and Schnepper (2021)	Y	Y	Y	Y	Y
Miselis et al. (2022)	Y	Y	Y	Y	Y
Lunde et al. (2022)	Y	Y	Y	CT	Y
Qualitative studies	Is the qualitative approach appropriate to answer the research question?	Are the qualitative data collection methods adequate to address the research question?	Are the findings adequately derived from the data?	Is the interpretation of results sufficiently substantiated by data?	Is there coherence between qualitative data sources, collection, analysis and interpretation?
Diniz et al. (2021)	Y	Y	Y	Y	Y

*Note:* CT, cannot tell; N, no; Y, yes.

## 3 Results

The search strategy was applied in January 2025 and returned a total of 981 records. After all analysis stages, 21 studies presenting 15 different tools were included in this scoping review (Figure 1).

### 3.1 Characteristics of the Sources of Evidence

The included articles (*n* = 21) were published between 2016 and 2024, with a higher concentration in 2020, 2021 and 2023 (*n* = 4 each). Ten articles were published in journals specifically focused on IPE and practice. Most of the articles were published in the United States (*n* = 10), followed by Indonesia (*n* = 4) and Japan (*n* = 3). Brazil, Canada, Norway, and Italy each had one publication. The general characteristics of the sources of evidence can be found in Table 2.

The purpose of most of the articles was to assess the effect and impact of interprofessional programmes by the IPC development in students (*n* = 8), followed by the description and evaluation of curricular models and educational structures (*n* = 4), analysis of collaboration in professional settings (*n* = 4), validation of tools for assessing IPC (*n* = 3) and evaluation of students' interprofessional attitudes and experiences during IPC development (*n* = 2).

The most cited professional categories in the articles were nursing (*n* = 17), medicine (*n* = 16) and pharmacy (*n* = 12). The scenarios cover a variety of primary care and community settings, with a focus on interprofessional care and services for family, community and vulnerable populations, varying according to the context of each country. These settings may be grouped into the following main categories: family health and primary care clinics and units (*n* = 10), community centres and programmes for underserved populations (*n* = 7), simulated environments and telehealth (*n* = 2), paediatric and dental care (*n* = 1) and geriatrics primary care (*n* = 1).

Among the 21 articles analysed, 15 assessment tools were cited, with one publication using two different tools [30]. The most frequently cited tools were original version of the Interprofessional Collaborative Competency Attainment Survey (ICCAS) (*n* = 5), a Japanese version of the Self‐assessment Scale of Interprofessional Competency (JASSIC) (*n* = 3) and the Interprofessional Education Collaborative (IPEC) Competency Self‐Assessment Survey (*n* = 2).

### 3.2 Quality Appraisal Results

The quality appraisal procedure using the MMAT framework revealed variations in the methodological quality of the 21 included studies. It is important to note that, although three of these articles were validation and translation studies, all were analysed using the same MMAT framework. Overall, 20 studies consistently met the two initial screening criteria related to the clarity of research questions and the adequacy of collected data to address them. The unique qualitative study met all MMAT criteria, while the quantitative and mixed‐methods designs showed specific methodological variations, as detailed in Table 4.

### 3.3 Characteristics of the Tools

Of the 15 assessment tools analysed, 13 were validated either by the authors or in previous studies, while two were specifically developed for their respective studies without undergoing formal psychometric validation [31, 32]. The number of items in each tool ranged from 9 to 42. The general characteristics of the tools can be found in Table 3.

#### 3.3.1 Evaluation Methods

All tools (*n* = 15) were designed for participants' self‐assessment of their IPC. The Indonesian version of the Interprofessional Collaborator Assessment Rubric (ICAR) may also be used by faculty for observational assessment of the competencies [33]. Almost all tools (*n* = 13) use the Likert or Likert‐type scales with agreement response options. Exceptions were the ICAR, which employs an assessment rubric with a series of columns for the dimensions, the numeric scale and behavioural indicators/performance criteria [33], and IPECC‐SET 38, which employs a self‐efficacy 100‐point rating scale. The developer of the latter argues that it provides a more refined way to assess sensitive indicators, avoiding the tendency to force extreme choices, which is often the case with a 5‐point scale [34].

#### 3.3.2 Theoretical Foundations

The theoretical frameworks and references on IPC used in the assessment tools (*n* = 15) were well‐grounded in the articles analysed or in the studies that developed the tools. The most commonly used frameworks for tool development were the Canadian Interprofessional Health Collaborative (CIHC) [30, 31, 33, 35, 36, 37, 38, 39, 40], the IPEC Core Competencies for IPCP [30, 32, 34, 41, 42, 43] and the IPC in Japan [44, 45, 46]. Another framework mentioned was the CanMEDS Competency Framework [47]. Three tools were developed based on findings from a previous qualitative study [48] and by conducting behavioural result interviews with high‐performing healthcare professionals [49, 50].

The authors of the ICCAS state that it originated from a set of competencies developed and validated in both English and French [51], but the same competencies are also reflected in the CIHC Competencies Framework. For the CHIBAS, competencies were derived from behavioural interviews and subsequently compared and validated against the domains and competencies of the IPEC framework and RIPLS instrument (Readiness for Interprofessional Learning) [52].

Although all the tools (*n* = 15) were applied in PHC settings, most were not originally developed specifically for this context. Exceptions include a few studies that, according to the authors, aimed for greater alignment with PHC: JASSIC was developed based on the IPC Framework in Japan to be applied in institutions and communities, as well as to learners and practitioners, particularly in the field of primary care [53]; the Indonesian Collaboration Competence Questionnaire (ICCQ) was primarily developed based on the literature and findings from a previous qualitative study with professionals working in PHC centres [48, 54]. Two other tools also aimed to reflect PHC needs but have not been validated: an instrument developed based on the IPEC Core Competencies, to examine changes in student knowledge related to interprofessional teamwork and public health competencies [33], and a questionnaire specifically developed for PHC professionals in Brazil that was based on the CIHC collaborative competencies [32].

#### 3.3.3 Domains and Competencies

The authors often indicate which domains and competencies are being assessed or the guiding framework from which they are derived. In general, these elements align with the following categories: *Roles and Responsibilities* (*n* = 15), *Teamwork and Collaboration* (*n* = 14), *Interprofessional Communication* (*n* = 12), *Patient, Family and Community‐Centred Care* (*n* = 10), *Values and Ethics* (*n* = 9), *Interprofessional Conflict Resolution* (*n* = 6), *Collaborative Leadership* (*n* = 5) and *Public Health Competencies* (*n* = 1).

A common characteristic of the tools not based on the most frequently used frameworks was the inclusion of competencies related to *Collaborative Leadership* [47, 48, 49, 50]. Although the CIHC framework has included the *Collaborative Leadership* competency since 2010, instruments derived from it have generally not incorporated this domain, with the exception of an instrument developed for the Brazilian context [31].

The questionnaire developed by Johansson et al., based on IPEC, incorporated public health competencies such as *Communication and Informatics*, *Epidemiology*, *Health Policy and Management* and *Social and Behavioural Sciences* [32]. Among the tools analysed, only the JASSIC and the Interprofessional Attitude Scale (IPAS), which were based on the IPEC framework and RIPLS (the Readiness for Interprofessional Learning) incorporated a community orientation through the competency *Patient, Family and Community‐Centred Care* [43, 44, 45, 46]. All interprofessional domains and competencies are described in Table 5.

**TABLE 5:** Domains and competencies assessed.

Assessment tool	Roles and responsibilities	Teamwork and collaboration	Interprofessional communication	Patient, family and community‐centred care	Ethics and values	Interprofessional conflict resolution	Collaborative leadership	Public health
Interprofessional Collaborative Competency Attainment Survey (ICCAS)								
Norwegian Version of the Interprofessional Collaborative Competency Attainment Survey (ICCAS)								
Interprofessional Collaborative Competency Attainment Survey‐Revised (ICCAS‐R)								
Untitled questionnaire by Diniz et al. (2021)								
Indonesian Version of the Interprofessional Collaborator Assessment Rubric (ICAR) instrument								
Interprofessional Education Collaborative (IPEC) Competency Self‐Assessment Survey								
Review of Interprofessional Competencies (RIPC)								
IPECC‐SET 38 tool								
Untitled questionnaire by Johansson et al. (2020)								
Interprofessional Attitude Scale (IPAS)								
Japanese Self‐Assessment Scale of Interprofessional Competency (JASSIC)								
Health Professional Collaborative Competency Perception Scale (HPCCPS)								
Indonesian Collaboration Competence Questionnaire (ICCQ)								
Italian Version of the Chiba Interprofessional Competency Scale (CICS29)								
Indonesian Version of the Chiba Interprofessional Competency Scale (CICS29)								

*Note:* The grey shaded cells indicate that a specific assessment tool (line) encompasses the corresponding competency (column). This shading visually maps which competencies are present in each instrument.

## 4 Discussion

The findings of this scoping review provide a comprehensive mapping of existing assessment tools for IPC within the PHC, offering insights into the diversity of evaluation methods, their theoretical foundations and the contexts in which they have been applied. By identifying 15 distinct tools across 21 studies, this review highlights the predominance of self‐assessment tools, suggesting a continued reliance on individual perception rather than objective and observational measures, which may influence the interpretation of competency development. Concerns have been expressed regarding the reliability of students or professionals in evaluating their own performance through self‐assessment tools [55].

Multiple methods can be used to assess key aspects of knowledge and competency development. Self‐assessment contributes significantly to the development of student competencies and positive learning outcomes [56], as it promotes increased self‐awareness and guides learners toward autonomous and reflective practice [57]. However, the predominance of self‐assessment tools identified in this review suggests a structural gap in the field, highlighting the need for more robust assessment strategies. Incorporating feedback from peers, faculty and patients, combined with structured reflection, can mitigate biases such as overconfidence and distorted self‐perception, particularly in early learners [58, 59]. Notably, only one tool identified in this review incorporated an observational component: the ICAR [33]. Considering the context of PHC, where interprofessional collaboration takes place within complex, context‐dependent and frequently unpredictable environments, workplace‐based assessment (WBA) is particularly valuable. By enabling direct observation, clinical case discussions and multisource feedback, WBA provides more accurate and context‐sensitive evaluation in real‐world settings. While various assessment forms can demonstrate theoretical knowledge, evidence suggests that competence alone does not reliably predict performance in clinical practice. Thus, a major advantage of WBA is its ability to evaluate professional delivery within the actual working environment [60, 61].

Most of the tools in this review included items measured using the Likert‐type scales. The tools vary considerably in the number of items and the breadth of domains included. While more concise instruments may facilitate implementation in busy PHC settings, they may also limit the comprehensiveness of the assessment. Conversely, tools with a broader range of domains can offer a more holistic view of IPC, although they may require more time and resources to apply.

The geographic distribution of studies emphasises significant contributions from the United States, Indonesia and Japan in developing, publishing and disseminating research on this topic, thereby advancing the field. The United States remains a predominant source of IPE studies, a trend reflecting the incorporation of IPE as an indicator of success within accreditation standards for health professions. Analyses demonstrate that 18 of 21 accreditation documents in the United States contain applicable IPE statements [62], with IPE in the curriculum identified as the most prevalent social mission indicator among accreditors [63]. Concurrently, there is a growing research focus in Asian countries, particularly Japan, where 71.9% of medical schools have implemented IPE programmes. This regional trend reflects robust national health education policies and cultural frameworks that inherently prioritise interprofessional collaboration [64, 65].

The fact that most publications occurred in 2020, 2021 and 2023 suggests a growing interest in IPE and collaborative practice research in recent years. This increase may be linked to the rising complexity of healthcare needs and contemporary challenges, such as the COVID‐19 pandemic, which highlighted the necessity for interprofessional collaboration in both education and health services [66, 67, 68].

Despite assessing a broad range of professional categories, the findings indicate a predominant focus on nursing, medicine and pharmacy. These results show both the fact that this field is well‐established in the professions mentioned [69, 70] and the need to strengthen research on the development and assessment of competencies in other professions within PHC settings.

An analysis of theoretical frameworks reveals the significant influence of IPEC and CIHC, underscoring their established role as key references for IPC and their contribution to ongoing development in the field. This consolidation is further evidenced by the predominant emphasis on fundamental dimensions of interprofessional practice, such as *Roles and Responsibilities*, *Teamwork and Collaboration* and *Interprofessional Communication* [19]. However, when considering PHC settings, the emergence of assessment tools tailored to specific contexts highlights the need for complementary approaches. The addition of community‐centred care [43, 44, 45, 46], leadership [47, 48, 49, 50] and public health [37] competencies in certain tools indicates an alignment with core capacities and challenges in PHC. This supports their potential applicability to the evolving demands of this specific context.

Considering that accessibility, continuity of care, comprehensiveness and coordination are central attributes of PHC [71], IPC required for effective practice in this area must be grounded in these foundational principles. Ensuring such alignment is essential for delivering continuous and integrated care.

The World Health Organization (WHO) framework emphasises the core competencies, for example, leadership, to be developed by healthcare workers to adequately engage in interprofessional teamwork. Collaborative leadership is characterised by shared decision‐making to achieve common goals, and it is crucial that every healthcare worker is able to act both as a team leader and as a team member. Moreover, the WHO affirms that individuals, families and communities should participate in their own care, acknowledging their uniqueness and self‐awareness in the process. Such participation may also serve to reduce inequalities [72].

The WHO also highlights the central role of social participation and community engagement in achieving universal health coverage and improving population health and well‐being, reiterating the importance of empowering individuals and communities as part of the PHC system [73]. This renewed emphasis calls for a revision of guiding frameworks, policies and assessment tools to explicitly incorporate community‐centred care as a core competency for professionals working in PHC settings.

Variations across the tools reveal the importance of critically selecting, adapting and validating assessment tools to ensure they respond effectively to both established frameworks and emerging demands in PHC.

PHC is a diverse concept with meanings and implementations that vary across contexts [71]. The unique ways in which each country conceptualises and organises PHC, along with the fact that IPE and IPCP are deeply influenced by national cultures and health systems, make it challenging to adopt standardised assessment instruments across different settings. Therefore, each local health system should develop or adapt evaluation methods that reflect their specific realities and priorities, ensuring alignment with national guidelines and cultural contexts.

Recent reviews have sought to identify tools for assessing collaborative competencies and understand their variations [20, 74], identifying interprofessional competency assessment tools used by educators [69] and self‐assessment or peer evaluation tools for students [75].

The unique contribution of this review lies in the selection of tools used to assess IPC in students or health professionals in PHC, allowing for the identification and standardisation of tools appropriate for this context. To facilitate their practical application, a User's Guide of the identified instruments was developed as a consultation resource for selection and implementation in PHC settings (Appendix A).

Most of the tools used to assess competencies in this field are not specifically designed for PHC, yet they are applied in this setting. These tools are capable of assessing essential competencies for teamwork that go beyond the specific ‘teamwork’ competency itself. A noteworthy feature of this review is that it includes validated tools not identified in previous studies [20, 69, 74, 75], which address other relevant core competencies for PHC, such as community‐centred care and coordination, management and leadership.

Previous studies have outlined the key competencies required for effective practice in this context. Shimizu and Fragelli highlight the importance of organising care, working collaboratively, conducting community diagnosis, planning community interventions, developing intersectoral actions, strengthening public policies, promoting educational initiatives with the health team and engaging with diverse groups [76]. Similarly, Michielsen et al. identify essential competencies for delivering person‐centred integrated care in PHC settings, including interprofessional communication, interprofessional collaborative teamwork, leadership and patient‐centred communication. Together, these contributions emphasise that strengthening competencies related to collaboration, community engagement and patient‐centredness is fundamental for advancing equitable, integrated and sustainable health systems [23].

### 4.1 Strengths and Limitations

As a scoping review, this study has inherent limitations. Although efforts were made to conduct a comprehensive search, it is possible that relevant studies were missed, particularly those published in nonindexed sources or in regions where the selected keywords are not the standard descriptors, potentially excluding significant local literature.

Given that PHC is organised in diverse ways across different health systems, shaped by political, cultural, social and organisational factors, it requires tools that are context‐sensitive and capable of capturing the specific demands of IPCP within each system. The present review plays an important role by mapping existing tools used internationally to assess IPC and provides a foundation for stakeholders to determine which tools to use or adapt according to the needs of different PHC settings.

## 5 Conclusion

This review offers a comprehensive overview of IPC assessment tools used in PHC settings. While most studies employ instruments originally developed to assess interprofessional collaboration in general, some tools, either adapted or created for particular health contexts, include domains that align with the principles of PHC, such as community‐centred care, collaborative leadership and public health competencies. Selecting an instrument that is appropriate for the specific context of use is essential, as it enhances the relevance and precision of the results, contributing more effectively to the development of IPE and collaborative practice in PHC.

## Author Contributions

**Ana Caroline Machado:** conceptualization, methodology, formal analysis, writing – original draft, writing – review and editing, data curation. **Alessandra da Silva Kulkamp:** methodology, formal analysis, writing – review and editing, conceptualization. **Ana Paula Rocha:** conceptualization, writing – original draft, formal analysis, methodology. **Ana Paula Veber:** conceptualization, writing – review and editing, formal analysis. **Fernanda Manzini:** conceptualization, writing – review and editing, formal analysis, supervision. **Isabel Cardoso de Carvalho:** conceptualization, writing – review and editing, formal analysis. **Mareni Rocha Farias:** conceptualization, writing – review and editing, formal analysis. **Marina Raijche Mattozo Rover:** conceptualization, writing – review and editing, formal analysis. **Marselle Nobre de Carvalho:** writing – review and editing, formal analysis. **Silvana Nair Leite:** conceptualization, writing – review and editing, supervision, formal analysis.

## Funding

This study was supported by the National Health Fund of the Brazilian Ministry of Health through the Decentralized Execution Term (TED 113/2023), and by the Coordination for the Improvement of Higher Education Personnel (CAPES). The funding agencies had no role in the design, execution, analysis or interpretation of the data.

## Ethics Statement

Due to the nature of the study, neither approval by the institutional review board nor informed consent was required.

## Conflicts of Interest

The authors declare no conflicts of interest.

## Supporting information


**Appendix S1:** Search strategies.

## Data Availability

The datasets used and analysed during the current study are available from the corresponding author on reasonable request.

## Appendix A

The choice of the ideal tool depends directly on the characteristics of the intervention and the desired assessment method.

For one‐off, short‐term interprofessional experiences, the ability to capture immediate changes is essential. Pre–post‐test tools with fewer items and a focused design are most suitable for reducing respondent fatigue. Examples include the HPCCPS (9 items), the questionnaire by Johansson et al. (2020) (15 items), the questionnaire by Diniz et al. (2021) (18 items) and the ICCAS and its variations (20 items).

For long‐term programmes, more robust and comprehensive instruments are required. They should be able to track the development of competencies over time and provide more detailed mapping of sub‐competencies. Examples include the CICS29 (29 items), the RIPC (30 items), the IPEC Competency Self‐Assessment Survey (42 items), the IPECC‐SET 38 (38 items), the ICCQ (28 items) and the IPAS (27 items).

Considering the context of PHC, the Japanese Self‐assessment Scale of Interprofessional Competency (JASSIC) consists of 18 items and is a tool specifically validated for the Primary Care setting by incorporating community orientation. The Indonesian version of the Interprofessional Collaborator Assessment Rubric (ICAR) stands out as a 29‐item instrument and is a competency‐based assessment rubric that can be used by faculty (preceptors) for direct observation or by students for self‐assessment.

## References

[1] A. G. N. Sena , J. Schutt‐Aine , J. Arenas , and S. Akaba , “Milestones on the Road to Health Equity at the Pan American Health Organization,” Revista Panamericana de Salud Pública 47 (2023): e42, 10.26633/rpsp.2023.42. 37123639 PMC10132698

[2] M. Marmot , “Health Equity in England: The Marmot Review 10 Years On,” BMJ Globalization and Health 368 (2020): m693, 10.1136/bmj.m693. 32094110

[3] C. Bouton , M. Journeaux , M. Jourdain , M. Angibaud , J.‐F. Huon , and C. Rat , “Interprofessional Collaboration in Primary Care: What Effect on Patient Health? A Systematic Literature Review,” BMC Primary Care 24, no. 1 (2023): 253, 10.1186/s12875-023-02189-0. 38031014 PMC10685527

[4] F. Vaseghi , M. Yarmohammadian , and A. Raeisi , “Interprofessional Collaboration Competencies in the Health System: A Systematic Review,” Iranian Journal of Nursing and Midwifery Research 27, no. 6 (2022): 496–504, 10.4103/ijnmr.ijnmr_476_21. 36712303 PMC9881554

[5] H. Wei , P. Horns , S. F. Sears , K. Huang , C. M. Smith , and T. L. Wei , “A Systematic Meta‐Review of Systematic Reviews About Interprofessional Collaboration: Facilitators, Barriers, and Outcomes,” Journal of Interprofessional Care 36, no. 5 (2022): 735–749, 10.1080/13561820.2021.1973975. 35129041

[6] S. Reeves , A. Xyrichis , and M. Zwarenstein , “Teamwork, Collaboration, Coordination, and Networking: Why We Need to Distinguish Between Different Types of Interprofessional Practice,” Journal of Interprofessional Care 32, no. 1 (2018): 1–3, 10.1080/13561820.2017.1400150. 29131697

[7] L. Smith , V. Johnson‐Lawrence , M. Andrews , and S. Parker , “Opportunity for Interprofessional Collaborative Care—Findings From a Sample of Federally Qualified Health Center Patients in the Midwest,” Public Health 151 (2017): 131–136, 10.1016/j.puhe.2017.07.009. 28797923

[8] E. Gucciardi , S. Espin , and A. Morganti , “Exploring Interprofessional Collaboration During the Integration of Diabetes Teams Into Primary Care,” BMC Family Practice 17, no. 12 (2016): 1–14, 10.1186/s12875-016-0407-1. 26831500 PMC4736701

[9] S. M. Smith , E. Wallace , B. Clyne , F. Boland , and M. Fortin , “Interventions for Improving Outcomes in Patients With Multimorbidity in Primary Care and Community Setting: A Systematic Review,” Systematic Reviews 10, no. 1 (2021): 1–271, 10.1186/s13643-021-01817-z. 34666828 PMC8527775

[10] S. Reeves , E. Clark , S. Lawton , M. Ream , and F. Ross , “Examining the Nature of Interprofessional Interventions Designed to Promote Patient Safety: A Narrative Review,” International Journal for Quality in Health Care 29, no. 2 (2017): 144–150, 10.1093/intqhc/mzx008. 28453828

[11] CIHC , CIHC Competency Framework for Advancing Collaboration (Canadian Interprofessional Health Collaborative, 2024).

[12] IPEC , IPEC Core Competencies for Interprofessional Collaborative Practice: Version 3 (Interprofessional Education Collaborative, 2023).

[13] S. Morgan , S. Pullon , and E. McKinlay , “Observation of Interprofessional Collaborative Practice in Primary Care Teams: An Integrative Literature Review,” International Journal of Nursing Studies 52, no. 7 (2015): 1217–1230, 10.1016/j.ijnurstu.2015.03.008. 25862411

[14] H. Almoghirah , H. Nazar , and J. Illing , “Assessment Tools in Pre‐Licensure Interprofessional Education: A Systematic Review, Quality Appraisal and Narrative Synthesis,” Medical Education 55, no. 7 (2021): 795–807, 10.1111/medu.14453. 33440040

[15] E. S. Anderson , “Evaluating Interprofessional Education: An Important Step to Improving Practice and Influencing Policy,” Journal of Taibah University Medical Sciences 11, no. 6 (2016): 571–578, 10.1016/j.jtumed.2016.08.012.

[16] C. Donnelly , R. Ashcroft , A. Mofina , N. Bobbette , and C. Mulder , “Measuring the Performance of Interprofessional Primary Health Care Teams: Understanding the Teams Perspective,” Primary Health Care Research & Development 20 (2019): e125, 10.1017/S1463423619000409. 31455458 PMC6719251

[17] P. O’Reilly , S. H. Lee , M. O’Sullivan , W. Cullen , C. Kennedy , and A. MacFarlane , “Assessing the Facilitators and Barriers of Interdisciplinary Team Working in Primary Care Using Normalisation Process Theory: An Integrative Review,” PLoS One 12, no. 5 (2017): e0177026, 10.1371/journal.pone.0177026. 28545038 PMC5436644

[18] G. D. Rogers , J. E. Thistlethwaite , E. S. Anderson , et al., “International Consensus Statement on the Assessment of Interprofessional Learning Outcomes,” Medical Teacher 39, no. 4 (2017): 347–359, 10.1080/0142159X.2017.1270441. 28024436

[19] J. E. Thistlethwaite , D. Forman , L. R. Matthews , G. D. Rogers , C. Steketee , and T. Yassine , “Competencies and Frameworks in Interprofessional Education: A Comparative Analysis,” Academic Medicine 89, no. 6 (2014): 869–875, 10.1097/ACM.0000000000000249. 24871237

[20] R. Allvin , C. Thompson , and S. Edelbring , “Variations in Measurement of Interprofessional Core Competencies: A Systematic Review of Self‐Report Instruments in Undergraduate Health Professions Education,” Journal of Interprofessional Care 38, no. 3 (2023): 486–498, 10.1080/13561820.2023.2241505. 37589390

[21] J. Peltonen , H. Leino‐Kilpi , H. Heikkilä , et al., “Instruments Measuring Interprofessional Collaboration in Healthcare–A Scoping Review,” Journal of Interprofessional Care 34, no. 2 (2020): 147–161, 10.1080/13561820.2019.1637336. 31331216

[22] Organization WH , Declaration of Astana. Global Conference on Primary Health Care Astana, Kazakhstan (WHO, 2018), https://www.who.int/publications/i/item/WHO‐HIS‐SDS‐2018.61.

[23] L. Michielsen , E. W. M. A. Bischoff , T. Schermer , and M. Laurant , “Primary Healthcare Competencies Needed in the Management of Personcentred Integrated Care for Chronic Illness and Multimorbidity: Results of a Scoping Review,” BMC Primary Care 24, no. 1 (2023): 98, 10.1186/s12875-023-02050-4. 37046190 PMC10091550

[24] N. R. Haddaway , M. J. Page , C. C. Pritchard , and L. A. McGuinness , “PRISMA2020: An R Package and Shiny App for Producing PRISMA 2020‐Compliant Flow Diagrams, With Interactivity for Optimised Digital Transparency and Open Synthesis,” Campbell Systematic Reviews 18, no. 2 (2022): e1230, 10.1002/cl2.1230. 36911350 PMC8958186

[25] M. D. J. Peters , C. Marnie , A. C. Tricco , et al., “Updated Methodological Guidance for the Conduct of Scoping Reviews,” JBI Evidence Implementation 19, no. 1 (2021): 3–10, 10.1097/XEB.0000000000000277. 33570328

[26] A. C. Tricco , E. Lillie , W. Zarin , et al., “PRISMA Extension for Scoping Reviews (PRISMA‐ScR): Checklist and Explanation,” Annals of Internal Medicine 169, no. 7 (2018): 467–473, 10.7326/M18-0850. 30178033

[27] M. Ouzzani , H. Hammady , Z. Fedorowicz , and A. Elmagarmid , “Rayyan—A Web and Mobile App for Systematic Reviews,” Systematic Reviews 5, no. 1 (2016): 210, 10.1186/s13643-016-0384-4. 27919275 PMC5139140

[28] Q. N. Hong , S. Fàbregues , G. Bartlett , et al., “The Mixed Methods Appraisal Tool (MMAT) Version 2018 for Information Professionals and Researchers,” Education for Information 34, no. 4 (2018): 285–291, 10.3233/EFI-180221.

[29] D. Pollock , E. L. Davies , M. D. Peters , et al., “Undertaking a Scoping Review: A Practical Guide for Nursing and Midwifery Students, Clinicians, Researchers, and Academics,” Journal of Advanced Nursing 77, no. 4 (2021): 2102–2113, 10.1111/jan.14743. 33543511 PMC8049063

[30] H. H. Miselis , S. Zawacki , S. White , et al., “Interprofessional Education in the Clinical Learning Environment: A Mixed‐Methods Evaluation of a Longitudinal Experience in the Primary Care Setting,” Journal of Interprofessional Care 36, no. 6 (2022): 845–855, 10.1080/13561820.2022.2025768. 35109762

[31] A. L. T. M. Diniz , R. H. V. D. Melo , and R. L. A. D. Vilar , “Análise de uma prática interprofissional colaborativa na Estratégia Saúde da Família,” Revista Ciência Plural 7, no. 3 (2021): 137–157, 10.21680/2446-7286.2021v7n3ID23953.

[32] P. Johansson , D. Nickol , S. Maloney , et al., “Health Professions Students' Assessment of an Interprofessional Rural Public Health‐Focused Rotation: A Pilot Study Based on the Community‐Oriented Primary Care Approach,” Journal of Medical Education and Curricular Development 7 (2020): 2382120520932549, 10.1177/2382120520932549. 32647748 PMC7325452

[33] A. B. T. Randita , W. Widyandana , and M. Claramita , “IPE‐COM: A Pilot Study on Interprofessional Learning Design for Medical and Midwifery Students,” Journal of Multidisciplinary Healthcare 12 (2019): 767–775, 10.2147/JMDH.S202522. 31571894 PMC6748318

[34] V. Gruss and M. Hasnain , “Building the Future Geriatrics Workforce Through Transformative Interprofessional Education and Community‐Engaged Experiential Learning,” Journal of Interprofessional Education & Practice 22 (2021): 100389, 10.1016/j.xjep.2020.100389.

[35] P. Chen , M. Clark , M. Sharma , Y. Troya , I. Cenzer , and J. Rivera , “A Longitudinal Workplace‐Based Interprofessional Curriculum for Graduate Learners in a Geriatrics Patient‐Centered Medical Home,” Journal of Interprofessional Education & Practice 24 (2021): 100459, 10.1016/j.xjep.2021.100459.

[36] E. H. Gilliam , W. Nuffer , J. M. Brunner , et al., “A Multimethod Evaluation of an Interprofessional IPPE in an Underserved Clinic,” Currents in Pharmacy Teaching and Learning 12, no. 6 (2020): 663–670, 10.1016/j.cptl.2020.01.036. 32482268

[37] E. Hartnett , J. Haber , P. Catapano , et al., “The Impact of an Interprofessional Pediatric Oral Health Clerkship on Advancing Interprofessional Education Outcomes,” Journal of Dental Education 83, no. 8 (2019): 878–886, 10.21815/JDE.019.088. 31010889

[38] L. Lunde , A. Moen , R. B. Jakobsen , B. Møller , E. O. Rosvold , and A. M. Brænd , “A Preliminary Simulation‐Based Qualitative Study of Healthcare Students' Experiences of Interprofessional Primary Care Scenarios,” Advances in Simulation 7, no. 1 (2022): 9, 10.1186/s41077-022-00204-5. 35314003 PMC8935844

[39] L. A. Waltz , J. N. Barnes , A. F. Crocker , et al., “Implementing a Primary Care Simulation to Improve Interprofessional Competencies,” Journal of Interprofessional Education & Practice 33 (2023): 100681, 10.1016/j.xjep.2023.100681.

[40] J. Zeien , J. Hanna , S. Yee , et al., “Education Without Walls: Using a Street Medicine Program to Provide Real‐World Interprofessional Learning,” Journal of Interprofessional Care 37, no. 1 (2023): 91–99, 10.1080/13561820.2021.2016663. 35015588

[41] A. M. Sevin , K. M. Hale , N. V. Brown , and J. W. McAuley , “Assessing Interprofessional Education Collaborative Competencies in Service‐Learning Course,” American Journal of Pharmaceutical Education 80, no. 2 (2016): 32, 10.5688/ajpe80232. 27073285 PMC4827583

[42] J. R. Timm and L. L. Schnepper , “A Mixed‐Methods Evaluation of an Interprofessional Clinical Education Model Serving Students, Faculty, and the Community,” Journal of Interprofessional Care 35, no. 1 (2021): 92–100, 10.1080/13561820.2019.1710117. 32013630

[43] D. Widyandana , “Evaluation Interprofessional Education Principle in a Longitudinal Community‐Based Program for 3 Schools of Health Professions: Medicine, Nursing, and Nutrition,” Journal of Pharmacokinetics and Pharmacodynamics 7, no. 1 (2018): 59, 10.22146/jpki.35553.

[44] R. Goto and J. Haruta , “Current Status of Interprofessional Competency Among Healthcare Professions in Japan: A Cross‐Sectional Web‐Based Survey,” Journal of General and Family Medicine 24, no. 2 (2023): 119–125, 10.1002/jgf2.601. 36909786 PMC10000264

[45] J. Haruta and R. Goto , “Factors Associated With Interprofessional Competencies Among Healthcare Professionals in Japan,” Journal of Interprofessional Care 37, no. 3 (2023): 473–479, 10.1080/13561820.2022.2099818. 35880788

[46] J. Haruta and R. Goto , “Exploring Factors Associated With Healthcare Professionals' Subjective Perceptions of Complex Issues in Primary Care in Japan: A Self‐Administered Survey Study on Confidence, Satisfaction and Burden Levels,” BMJ Open 14, no. 3 (2024): e081328, 10.1136/bmjopen-2023-081328. PMC10966809 38531578

[47] J. Peranson , C. A. Weis , M. Slater , J. Plener , and D. Kopansky‐Giles , “An Interprofessional Approach to Collaborative Management of Low‐Back Pain in Primary Care: A Scholarly Analysis of a Successful Educational Module for Prelicensure Learners,” Journal of Chiropractic Education 38, no. 1 (2024): 30–37, 10.7899/JCE-22-24. 38329313 PMC11097224

[48] D. K. Ernawati , N. K. Sutiari , I. W. Astuti , H. Onishi , and B. Sunderland , “Correlation Between Intercultural Sensitivity and Collaborative Competencies Amongst Indonesian Healthcare Professionals,” Journal of Interprofessional Education & Practice 29 (2022): 100538, 10.1016/j.xjep.2022.100538.

[49] D. Soemantri , S. P. Sari , T. Wahyuni , et al., “Measuring the Interprofessional Collaborative Competencies of Health‐Care Students Using a Validated Indonesian Version of the CICS29,” Journal of Interprofessional Care 34, no. 6 (2020): 763–771, 10.1080/13561820.2019.1697215. 31829770

[50] A. Tonarelli , T. Yamamoto , C. Foà , et al., “Italian Validation of the Chiba Interprofessional Competency Scale (CICS29),” Acta Bio‐Medica 91, no. Suppl 2 (2020): 58–66, 10.23750/abm.v91i2-S.9172. 32168314 PMC7944661

[51] V. Curran , L. Casimiro , V. Banfield , et al., “Research for Interprofessional Competency‐Based Evaluation (RICE),” Journal of Interprofessional Care 23, no. 3 (2009): 297–300, 10.1080/13561820802432398. 19242851

[52] T. Yamamoto , I. Sakai , Y. Takahashi , T. Maeda , Y. Kunii , and K. Kurokochi , “Development of a New Measurement Scale for Interprofessional Collaborative Competency: A Pilot Study in Japan,” Journal of Interprofessional Care 28, no. 1 (2014): 45–51, 10.3109/13561820.2013.851070. 24180512

[53] J. Haruta and R. Goto , “Development of a Japanese Version of the Self‐Assessment Scale of Interprofessional Competency (JASSIC),” Journal of Interprofessional Care 36, no. 4 (2021): 599–606, 10.1080/13561820.2021.1951188. 34355655

[54] D. K. Ernawati , “Collaborative Competencies in Public Health Center in Indonesia: An Explorative Study,” Journal of Interprofessional Education & Practice 18 (2020): 100299, 10.1016/j.xjep.2019.100299.

[55] N. Yates , S. Gough , and V. Brazil , “Self‐Assessment: With All Its Limitations, Why Are We Still Measuring and Teaching It? Lessons From a Scoping Review,” Medical Teacher 44, no. 11 (2022): 1296–1302, 10.1080/0142159x.2022.2093704. 35786121

[56] C. M. Cheong , N. Luo , X. Zhu , Q. Lu , and W. Wei , “Self‐Assessment Complements Peer Assessment for Undergraduate Students in an Academic Writing Task,” Assessment & Evaluation in Higher Education 48, no. 1 (2023): 135–148, 10.1080/02602938.2022.2069225.

[57] S. Kajander‐Unkuri , H. Leino‐Kilpi , J. Katajisto , et al., “Congruence Between Graduating Nursing Students' Self‐Assessments and Mentors' Assessments of Students' Nurse Competence,” Collegian 23, no. 3 (2016): 303–312, 10.1016/j.colegn.2015.06.002.

[58] S. A. Pawluk , M. Zolezzi , and D. Rainkie , “Comparing Student Self‐Assessments of Global Communication With Trained Faculty and Standardized Patient Assessments,” Currents in Pharmacy Teaching and Learning 10, no. 6 (2018): 779–784, 10.1016/j.cptl.2018.03.012. 30025780

[59] S. N. Taylor , “Student Self‐Assessment and Multisource Feedback Assessment: Exploring Benefits, Limitations, and Remedies,” Journal of Management Education 38, no. 3 (2014): 359–383, 10.1177/105256291348.

[60] H. L. Anderson , J. Kurtz , and D. C. West , “Implementation and Use of Workplace‐Based Assessment in Clinical Learning Environments: A Scoping Review,” Academic Medicine 96 (2021): S164–S174, 10.1097/ACM.0000000000004366. 34406132

[61] A. Miller and J. Archer , “Impact of Workplace Based Assessment on Doctors' Education and Performance: A Systematic Review,” BMJ 341 (2010): 341, 10.1136/bmj.c5064. PMC2945627 20870696

[62] J. Zorek and C. Raehl , “Interprofessional Education Accreditation Standards in the USA: A Comparative Analysis,” Journal of Interprofessional Care 27, no. 2 (2013): 123–130, 10.3109/13561820.2012.718295. 22950791

[63] J. Orban , C. Xue , S. Raichur , et al., “The Scope of Social Mission Content in Health Professions Education Accreditation Standards,” Academic Medicine 97, no. 1 (2022): 111–120, 10.1097/ACM.0000000000004437. 34618736

[64] B. Lee , K. Phrasisombath , H. Shinozaki , et al., “Key Factors Affecting the Integration of Interprofessional Education Into Human Resources for Health Reform: A Lao People's Democratic Republic Case Study,” Journal of Interprofessional Care 33, no. 4 (2019): 356–360, 10.1080/13561820.2019.1646229. 31431108

[65] T. Maeno , J. Haruta , A. Takayashiki , H. Yoshimoto , R. Goto , and T. Maeno , “Interprofessional Education in Medical Schools in Japan,” PLoS ONE 14, no. 1 (2019): e0210912, 10.1371/journal.pone.0210912. 30653563 PMC6336262

[66] A. D. C. Belarmino , M. E. N. G. Rodrigues , S. D. J. S. B. D. Anjos , and A. R. Ferreira Júnior , “Collaborative Practices From Health Care Teams to Face the Covid‐19 Pandemic,” Revista Brasileira de Enfermagem 73 (2020): e20200470, 10.1590/0034-7167-2020-0470. 33111780

[67] S. F. Fernandes , J. G. Trigueiro , M. A. F. Barreto , et al., “Interprofessional Work in Health in the Context of the COVID‐19 Pandemic: A Scoping Review,” Revista da Escola de Enfermagem da USP 55 (2021): e20210207, 10.1590/1980-220x-reeusp-2021-0207. 34807228

[68] J. Frenk , L. C. Chen , L. Chandran , et al., “Challenges and Opportunities for Educating Health Professionals After the COVID‐19 Pandemic,” Lancet 400, no. 10362 (2022): 1539–1556, 10.1016/S0140-6736(22)02092-X. 36522209 PMC9612849

[69] S. Brownie , D. Blanchard , I. Amankwaa , et al., “Tools for Faculty Assessment of Interdisciplinary Competencies of Healthcare Students: An Integrative Review,” Frontiers in Medicine 10 (2023): 1124264, 10.3389/fmed.2023.1124264. 37396887 PMC10314362

[70] C. Rawlinson , T. Carron , C. Cohidon , et al., “An Overview of Reviews on Interprofessional Collaboration in Primary Care: Barriers and Facilitators,” International Journal of Integrated Care 21, no. 2 (2021): 32, 10.5334/ijic.5589. 34220396 PMC8231480

[71] M. Akman , “Primary Health Care From the Global Perspective: Lessons Learned From Current Research,” Primary Health Care Research & Development 18, no. 3 (2017): 209–211, 10.1017/S1463423617000226. 28463100

[72] Organization PAH , Interprofessional Health Teams for Integrated Care (PAHO, 2025), 10.37774/9789275129791.

[73] Organization WH , Social Participation for Universal Health Coverage, Health and Well‐Being (WHO, 2024), https://apps.who.int/gb/ebwha/pdf_files/WHA77/A77_ACONF3‐en.pdf.

[74] C. C. A. D. Oliveira , M. G. D. F. Morais , H. F. D. Cunha , et al., “Instrumentos de avaliação de competências colaborativas na educação interprofissional: Revisão integrativa da literatura,” Educação e Pesquisa 50 (2024): e269950, 10.1590/s1678-4634202450269950.

[75] S. Brownie , J. R. Yap , D. Blanchard , et al., “Tools for Self‐ or Peer‐Assessment of Interprofessional Competencies of Healthcare Students: A Scoping Review,” Front Med (Lausanne) 11 (2024): 1449715, 10.3389/fmed.2024.1449715. 39421863 PMC11483372

[76] H. E. Shimizu and T. B. O. Fragelli , “Competências Profissionais Essenciais para o Trabalho no Núcleo de Apoio à Saúde da Família,” Revista Brasileira de Educação Médica 40, no. 2 (2016): 216–225, 10.1590/1981-52712015v40n2e02702014.

