# Supplementary material for: Assessment of Interprofessional Competencies in Primary Health Care: A Scoping Review
Source: Clin Teach. 2026 Aug 13;23(5):e70491. doi: 10.1111/tct.70491 (PMC13473816; doi:10.1111/tct.70491)
Supplement: Supplementary file 1 [file TCT-23-e70491-s001.docx]

Supplementary Appendix A. Search Strategies

| **Databases** | **Search strategies** |
| --- | --- |
| **Pubmed** | ("core competenc*"[Title/Abstract] OR "interprofessional competenc*"[Title/Abstract] OR "collaborative competenc*"[Title/Abstract]) AND ("assessment*"[Title/Abstract] OR "evaluation"[Title/Abstract] OR "measurement"[Title/Abstract] OR "tool"[Title/Abstract] OR "instrument"[Title/Abstract] OR "scales"[Title/Abstract] OR "scaling"[Title/Abstract] OR "test*"[Title/Abstract] OR "rate"[Title/Abstract] OR "ratings"[Title/Abstract] OR "survey*"[Title/Abstract] OR "questionnaire*"[Title/Abstract]) AND ("Primary Health Care"[MeSH Terms] OR "Primary Health Care"[All Fields] OR "Primary Healthcare"[All Fields] OR "Primary Care"[All Fields]) |
| **Embase** | ('core competenc*':ti,ab,kw OR 'interprofessional competenc*':ti,ab,kw OR 'collaborative competenc*':ti,ab,kw) AND ('assessment*':ti,ab,kw OR 'evaluation':ti,ab,kw OR 'measurement':ti,ab,kw OR 'tool':ti,ab,kw OR 'instrument':ti,ab,kw OR 'scales':ti,ab,kw OR 'scaling':ti,ab,kw OR 'test*':ti,ab,kw OR 'rate':ti,ab,kw OR 'ratings':ti,ab,kw OR 'survey*':ti,ab,kw OR 'questionnaire*':ti,ab,kw) AND ('primary health care'/exp OR 'primary health care' OR 'primary healthcare' OR 'primary care') |
| **CINAHL** | ("core competenc*" OR "interprofessional competenc*" OR "collaborative competenc*") AND (assessment* OR evaluation OR measurement OR tool OR instrument OR scales OR scaling OR test* OR rate OR ratings OR survey* OR questionnaire*) AND ("Primary Health Care" OR "Primary Healthcare" OR "Primary Care") |
| **Scopus** | TITLE-ABS-KEY("core competenc*" OR "interprofessional competenc*" OR "collaborative competenc*") AND TITLE-ABS-KEY(assessment* OR evaluation OR measurement OR tool OR instrument OR scales OR scaling OR test* OR rate OR ratings OR survey* OR questionnaire*) AND TITLE-ABS-KEY("Primary Health Care" OR "Primary Healthcare" OR "Primary Care") |
| **Web of Science** | TS=("core competenc*" OR "interprofessional competenc*" OR "collaborative competenc*") AND TS=(assessment* OR evaluation OR measurement OR tool OR instrument OR scales OR scaling OR test* OR rate OR ratings OR survey* OR questionnaire*) AND TS=("Primary Health Care" OR "Primary Healthcare" OR "Primary Care") |
| **Lilacs** | TS=("core competenc*" OR "interprofessional competenc*" OR "collaborative competenc*") AND TS=(assessment* OR evaluation OR measurement OR tool OR instrument OR scales OR scaling OR test* OR rate OR ratings OR survey* OR questionnaire*) AND TS=("Primary Health Care" OR "Primary Healthcare" OR "Primary Care") |
| **Scielo** | ("core competenc*" OR "interprofessional competenc*" OR "collaborative competenc*" OR "competências interprofissionais" OR "competências colaborativas" OR "habilidades interprofesional*" OR "habilidades colaborativas") AND (assessment* OR evaluation OR measurement OR tool OR instrument OR scales OR scaling OR test* OR rate OR ratings OR survey* OR questionnaire* OR instrumentos OR instrumento OR ferramenta OR escalas OR teste OR testes OR cuestionarios OR questionários OR avaliação OR evaluación) AND ("Primary Health Care" OR "Primary Healthcare" OR "Primary Care" OR "Atenção Básica à Saúde" OR "Atención Primaria de Salud") |
| **Google acadêmico** | ("core competencies" OR "core competence" OR "interprofessional competence" OR "collaborative competence" OR "collaborative competencies") AND (assessment OR evaluation OR measurement OR tool OR instrument OR scales OR test OR rate OR ratings OR survey OR questionnaire OR instrumento OR ferramenta OR escala OR teste OR cuestionarios OR questionários OR avaliação OR evaluación) AND ("Primary Health Care" OR "Primary Healthcare" OR "Primary Care" OR "Atenção Básica à Saúde" OR "Atención Primaria de Salud") |
